# Operation for Double Hare-Lip, in an Adult

**Published:** 1838-03-14

**Authors:** Thomas Harris

**Affiliations:** Surgeon to the Hospital


*Operation for double Hare-Lip, in an adult. By* Thomas Harris, M. D., *Surgeon to the Hospital.*

Francis Farrel, an Irishman, 25 years of age, was admitted into the hospital 10th February, for this congenital malformation.

It was hereditary, his mother having had a single fissure, extending through the soft and hard palate.

*Wednesday, 14th February.* Dr. Harris performed the operation on both sides at the same time. He used a pair of scissors, in cutting the edges of the lip. Two long silver needles were then passed through the three portions of the lip, which were retained in their place, by the usual ligature.

Two days after the operation, erysipelas, and some sloughing of the lower edges of the incisions, on the inner side, took place. On the third day, hemorrhage, to the amount of three ounces, came on, which was checked by the application of dry lint. This was repeated several times, weakening the man so much, as to require the exhibition of stimuli. The pins were removed, one on the fourth, the other on the fifth day, and the man was discharged on 3d March, seventeen days after the operation. Owing to the sloughing, there still remained two fissures, of about two lines in depth. The man declined remaining for further relief.

